# Multiplexed Internet of Things Data Transmission and Visualization Utilizing Wireless LAN Authentication and Privacy Infrastructure Protocol in Smart Factories

**DOI:** 10.3390/s25103134

**Published:** 2025-05-15

**Authors:** Peng Zhang, Yanhong Yang, Yili Zheng, Yizhen Sun

**Affiliations:** 1 School of Technology, Beijing Forestry University, Beijing 100083, China; zhangpeng@sparksec.cn; 2 School of Information Engineering, Beijing Institute of Graphic Communication, Beijing 102600, China; yangyanhong@bigc.edu.cn; 3 State Grid Information and Communication Company of Hunan Electric Power Corporation, Changsha 410029, China; svnshine511@163.com

**Keywords:** data security transmission, WAPI protocol, smart factories

## Abstract

In high-security environments, such as smart factories and power networks, secure and reliable data transmission and device access are crucial. This paper proposes a diversified Internet of Things (IoT) data transmission and visualization solution based on the Wireless LAN Authentication and Privacy Infrastructure (WAPI) protocol. First, we introduce the technical characteristics of the WAPI protocol and analyze network access solutions relevant to smart factories, along with their application requirements in power networks. Addressing gaps in the existing literature, we present a comprehensive solution that encompasses network deployment, device management, device testing, and data transmission, highlighting its completeness and effectiveness in real-world applications. Furthermore, we provide detailed experimental evidence demonstrating the WAPI protocol’s effectiveness and security in supporting multiple terminal device access within high-security settings. The findings of this research not only fill critical gaps in existing work but also provide valuable technical support and reference for secure data management and real-time monitoring in smart-factory environments.

## 1. Introduction

In the era of Industry 4.0, the deployment of securely interconnected smart networks has become a fundamental requirement for various industries, notably in power grids and smart factories. These networks facilitate real-time data collection, monitoring, and analysis, thus improving operational efficiency, enabling predictive maintenance and informing decision-making processes. However, significant challenges are presented by the increasing integration of diverse devices and the complexity of data transmission, particularly in the realms of network security and data integrity. For example, Priyanka et al. proposed a framework for IoT-based smart-factory applications that emphasized security issues related to data transmission and device management [1]. Similarly, Wang et al. investigated challenges and solutions related to data interoperability and security in smart grids, highlighting the need for robust network protocols [2].

Recognizing the need for enhanced security in smart factories, the State Grid has begun deploying the WAPI protocol, a Chinese national standard for wireless networks designed to provide robust security features. WAPI incorporates advanced cryptographic algorithms, including elliptic curve cryptography (ECC) and symmetric encryption schemes. These includes strong encryption and mutual authentication, making WAPI particularly well suited for the high-security demands of power grids and industrial applications. WAPI was adopted as ISO/IEC 29167-16:2022, which was published as “Information technology—Automatic identification and data capture techniques—Part 16: Crypto suite ECDSA-ECDH security services for air interface communications” [3].

In response to these demands, we propose a unified solution based on the WAPI protocol that supports diversified data transmission. Our approach ensures seamless and secure communication across various devices within smart factories and the power grid. Additionally, our solution supports remote data visualization, allowing users to monitor and analyze data in real time from any location, thus facilitating informed decision making and proactive management of industrial operations. Previous studies have addressed various aspects of data security and visualization and proposed a secure visualization framework for IoT data [4]. However, our approach distinguishes itself by leveraging the unique security features of the WAPI protocol to ensure data integrity and confidentiality in various industrial applications.

The remainder of the paper is organized as follows: Section 2 summarily discusses the related work to emphasize the practical needs of the work carried out; Section 3 describes the data transmission of devices and implementation using WAPI; Section 4 shows the test results and verification, including the high-definition camera, sensors, and helmet; Section 5 summarizes and offers insights into the work presented in this paper.

## 2. Related Work

The integration of secure communication, efficient data transmission, and advanced visualization technologies in industrial and critical infrastructure environments has garnered significant academic interest. However, much of the existing literature remains domain-specific and lacks unified solutions adaptable to the dynamic demands of modern smart-factory systems.

### 2.1. Secure Communication Architectures in Industrial IoT

Li et al. proposed a trust-chain-based RFID architecture that introduced layered management and innovative authentication protocols to verify tag authenticity without third-party certification [5]. While this work enhanced security in RFID systems, it was narrowly scoped and lacked generalizability across heterogeneous industrial devices. Similarly, Priyanka et al. developed a portable IoT module for oil pipeline monitoring with integrated online data communication and web-based analytics [6]. Although effective for real-time monitoring, it fell short in addressing broader interoperability and multi-protocol integration challenges faced in diverse smart-factory environments.

Fan et al. introduced WAPI-based communication for securing smart distribution transformers, focusing on grid applications [7]. However, their implementation was limited to a specific subset of devices. In contrast, our work broadens the applicability of WAPI, proposing a unified scheme for both OS and non-OS industrial devices within complex factory environments.

### 2.2. Multicast Communication and Network Efficiency

Kumar’s multicast scheme focused on reducing computational and communication overhead while ensuring robust security [8]. Although valuable for efficiency, it did not integrate with protocol-specific constraints such as WAPI or address device heterogeneity. Our solution incorporates multicast optimization directly into WAPI configurations without compromising interoperability or security.

### 2.3. IoT and Edge Computing Integration

Mustafa et al. proposed a cross-layer, secure, and energy-efficient IoT framework that emphasized multilayered security protocols [9]. While it contributed to the energy–security trade-off, it lacked integration with standardized protocols like WAPI. Li et al. introduced a natural actor–critic method to optimize service chaining via edge intelligence [10], which improved adaptability but did not consider protocol-level security mechanisms in industrial communication.

### 2.4. Wireless Technologies in Smart Manufacturing

Noor-A-Rahim et al. and Seferagić et al. reviewed wireless technologies for smart manufacturing, highlighting trade-offs between latency, reliability, and security [11,12]. These surveys identified challenges but did not propose actionable implementations. Our work directly addressed these trade-offs through a lightweight, protocol-embedded approach that maintained both efficiency and security.

### 2.5. Data Visualization and Middle Platforms

Visualization tools are essential for industrial decision-making. Existing works have evaluated power-grid visualizations and proposed enterprise data middle platforms to support unified monitoring and interoperability [13,14]. However, these are often decoupled from the communication infrastructure. Our implementation tightly integrates visualization with secure WAPI-based data acquisition, enabling real-time feedback and unified control.

### 2.6. Comparative Insight

Despite the breadth of existing research, few studies provide a comprehensive end-to-end implementation that simultaneously addresses secure communication, heterogeneous device compatibility, and operational data visualization. Our work distinguishes itself by proposing a holistic framework based on the WAPI protocol, which is thoroughly validated in real-world industrial environments. This framework not only ensures the security of various device types but also effectively bridges the longstanding gap between communication infrastructure and real-time data visualization—an essential requirement often neglected in prior studies.

## 3. A Diversified IoT Data Transmission Based on WAPI

In this section, we first outline the access process standards of WAPI, providing a comprehensive overview of the procedures involved in establishing secure connections. Following this, we elaborate on the WAPI data packet structure, highlighting the essential components for secure communication. Subsequently, we present access strategies for various types of devices, demonstrating the flexibility and adaptability of the WAPI protocol in diverse industrial environments. Finally, we conduct a theoretical analysis of the protocol’s security to reinforce its robustness in wireless communication scenarios.

### 3.1. Protocol Authentication Process

**Figure 1:**
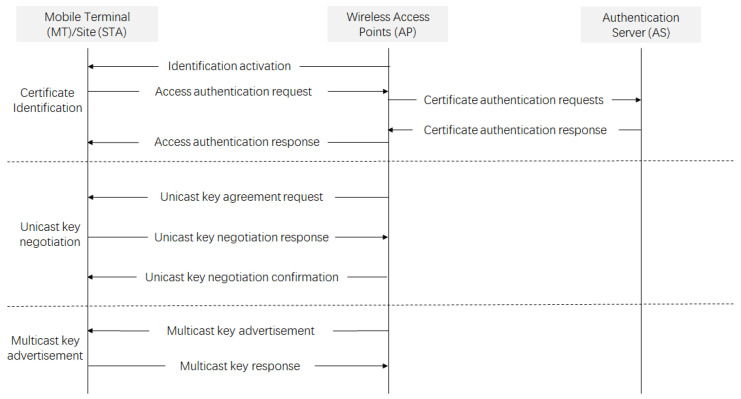
WAPI authentication access process.

The WAPI protocol establishes a robust security framework for wireless communication, specifically designed to meet the stringent security requirements of diverse application scenarios. The process for a WAPI terminal to access a WAPI wireless network is depicted in the following Figure 1. It involves three main steps: certificate authentication, unicast key negotiation, and multicast key announcement. The WAPI management layer implements wireless access control, ensuring that only authorized devices can connect to the wireless network and that devices connect to a trusted wireless network. They are described as follows:

- Certificate verification: This step involves mutual authentication between the terminal device (STA) and the access point (AP). Initially, an authentication request is sent by the STA to the AP, which includes its digital certificate. Upon receiving the request, a certificate validation request is forwarded by the AP to the Authentication Server (AS). The validity of the certificates from both the STA and the AP is verified by the AS. Based on the verification results, feedback is provided to the AP by the AS, which then decides whether to allow the STA to access the network.
- Unicast key negotiation: Following successful certificate verification, a unicast key is negotiated between the STA and AP. This key is critical for encrypting communication between the STA and AP, ensuring the confidentiality and integrity of the transmitted data. The unicast key is unique to each STA–AP pair, enhancing security by isolating communication sessions from one another.
- Multicast key distribution: The AP generates a multicast key and securely distributes it to authenticated devices. This key is used to encrypt broadcast and multicast data, preventing unauthorized access.

### 3.2. WAPI Data Packet Structure

**Figure 2:**
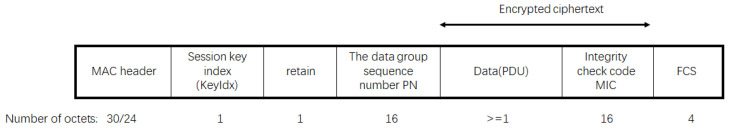
WAPI data packet structure.

After the key exchange, AP and STA can securely transmit unicast and multicast data. WAPI’s data packet structure includes several fields specifically designed for security. Figure 2 illustrates the WAPI data packet structure. The Wireless Header contains essential information about the data packet, including the source and destination addresses and the Packet Number, which is crucial for maintaining the order and uniqueness of the packets. The Data Payload encompasses the actual data being transmitted, which are encrypted using the session keys established during the key negotiation phase, ensuring confidentiality.

In addition, the Message Integrity Code (MIC) is computed on the data payload and part of the wireless header, ensuring data integrity and authenticity. The MIC is encrypted and transmitted with the data, enabling the receiver to verify that the data have not been tampered with, thus protecting against unauthorized alterations and ensuring the integrity of the communication.

### 3.3. Diversified IoT Device Terminal Access Solutions

#### 3.3.1. Devices with Operating Systems

Devices equipped with operating systems, such as Windows PCs, have more flexibility and capability in managing digital certificates required for secure communication over WAPI networks. P12 and P10 for certificate distribution are used, each with its unique mechanisms to ensure the secure installation and management of certificates on these devices.

##### P12 Certificate Distribution

Devices receive P12-format WAPI certificates by installing the appropriate WAPI driver from Intel or other vendors. The P12 format (PKCS 12) serves as a secure container format to store digital certificates, including public and private keys. When the P12 certificate is generated, a password is set to encrypt the certificate file, which has to be provided during the certificate installation on the device. This method ensures that the certificate is securely distributed and stored on the device, preventing unauthorized access.

##### P10 Certificate Request Process

This method involves the generation of a pair of keys (public and private keys) on the terminal or AP. The device then creates a certificate signing request (CSR), known as a P10 file, which contains the public key and device information but excludes the private key. The P10 file is sent to the AS, which generates a public key certificate and sends it back to the requesting device. The private key remains securely stored on the device, ensuring that it is never exposed during the request or distribution process. This method provides a high level of security by preventing the private key from being transmitted over the network.

#### 3.3.2. Devices Without Operating Systems

For devices that lack operating systems or have limited connectivity options, ensuring secure communication becomes more challenging due to their restricted capabilities. For instance, devices that rely on RS232 or RS485 interfaces are commonly used in industrial and automation environments due to their simplicity, robustness, and long-range communication capabilities.

These devices utilize the Modbus RTU (Remote Terminal Unit) protocol, a widely adopted serial communication standard for industrial automation, to facilitate reliable data transmission between master and slave devices. Data collected by these devices are sent in real-time to a Customer-Premises Equipment (CPE) device equipped with a WAPI certificate. The CPE device manages one or multiple sensor devices, ensuring centralized and secure data transmission to the network. This setup proves particularly useful in industrial environments where robust physical connections and data integrity are crucial.

In enclosed environments such as factories, physical isolation is employed to verify and protect data. This method involves isolating the data from external networks, thereby reducing the risk of unauthorized access and ensuring that data collected on-site are securely transmitted and processed. This approach is effective in maintaining data integrity and security in highly controlled environments.

### 3.4. Theoretical Analysis of WAPI Protocol Security

The WAPI protocol incorporates robust security mechanisms, including certificate verification, unicast key negotiation, and multicast key distribution. Each step is designed to protect the communication from unauthorized access, ensuring both confidentiality and integrity. This section presents the key features and mechanisms of WAPI, followed by an analysis of its security architecture.

#### 3.4.1. Certificate Authentication and Verification

The certificate verification process in WAPI relies on SM2 for elliptic curve cryptography to ensure secure mutual authentication between the terminal device’s (STA) and access point (AP). The probability of an attacker successfully forging a certificate is negligible due to the strength of the SM2 algorithm and the secure Public Key Infrastructure (PKI) system used for verification.

Let $C_{\mathrm{STA}}$ and $C_{\mathrm{AP}}$ represent the certificates of the STA and AP, respectively. The verification functions $V_{\mathrm{STA}}\left(C_{\mathrm{STA}}\right)$ and $V_{\mathrm{AP}}\left(C_{\mathrm{AP}}\right)$ are defined as follows:

(1) $$V_{\mathrm{STA}}\left(C_{\mathrm{STA}}\right)=\mathrm{True}\;\mathrm{if}\;\mathrm{STA}’s\;\mathrm{certificate}\;\mathrm{was}\;\mathrm{valid}$$

(2) $$V_{\mathrm{AP}}\left(C_{\mathrm{AP}}\right)=\mathrm{True}\;\mathrm{if}\;\mathrm{AP}’s\;\mathrm{certificate}\;\mathrm{was}\;\mathrm{valid}$$

The probability of certificate forgery, denoted by $P_{\mathrm{forgery}}$, is given by:

(3) $$P_{\mathrm{forgery}}\approx \frac{1}{2^{256}}\;\mathrm{for}\;\mathrm{SM}2\;\mathrm{with}\;256\text{-bit}\;\mathrm{security}$$

Thus, the probability of a successful certificate forgery is extremely low.

The certificate verification process acts as the first layer of security in WAPI, ensuring mutual authentication between the STA and AP through digital certificates.

In a secure PKI system, the probability of successfully forging a certificate is negligible due to the strength of the underlying cryptographic algorithms. Therefore, the probability of a successful forgery, $P_{\mathrm{forgery}}$, is defined as:

(4) $$P_{\mathrm{forgery}}=1-P_{\mathrm{secure}}$$

where $P_{\mathrm{secure}}$ is the probability that the certificate is correctly verified. In a secure PKI system (such as RSA with 2048-bit keys), $P_{\mathrm{secure}}$ approaches one, making $P_{\mathrm{forgery}}$ practically zero:

(5) $$P_{\mathrm{forgery}}\approx \frac{1}{2^{2048}}\;(\mathrm{practically}\;\mathrm{zero})$$

Therefore, the certificate verification process ensures a very high level of security against impersonation attacks.

**Theorem** **1.** *If both the STA and AP present valid certificates, and the Authentication Server (AS) validates these certificates using a secure PKI system, then the risk of an attacker impersonating either party is negligible.*

**Proof.**  Let *A* represent an attacker attempting to present a forged certificate to the AS. For the attack to succeed, *A* would need to possess the private key associated with the certificate of either the STA or the AP. Given the computational difficulty of private key recovery from a public key, especially using RSA’s intractability, the probability of this event occurring is exceedingly small. Thus, with high probability, $V_{\mathrm{STA}}\left(C_{\mathrm{STA}}\right)=\mathrm{True}$ and $V_{\mathrm{AP}}\left(C_{\mathrm{AP}}\right)=\mathrm{True}$ for valid certificates. □

#### 3.4.2. Unicast Key Negotiation

Once certificate verification has completed, a secure unicast key is negotiated between the STA and AP. This key is used to encrypt communication between the two parties.

Let $K_{\mathrm{unicast}}$ represent the shared secret between the STA and AP. The encryption of a message $P_{\mathrm{data}}$ using the unicast key is given by:

(6) $$C_{\mathrm{data}}={\mathrm{Enc}}_{K_{\mathrm{unicast}}}\left(P_{\mathrm{data}}\right)$$

where Enc is the encryption function that applied the unicast key.

The unicast key $K_{\mathrm{unicast}}$ can be compromised if an attacker successfully guesses the key. The strength of the key is crucial for the security of this process.

Let $P_{\mathrm{break}}$ represent the probability that an attacker can break the unicast encryption. For RSA-based encryption, the probability of a successful brute-force attack is related to the number of bits in the key, denoted by *n*. The probability of breaking the unicast key using a brute-force attack is:

(7) $$P_{\mathrm{break}}\approx \frac{1}{2^{n}}$$

For a 128-bit AES key, the probability is:

(8) $$P_{\mathrm{break}}\approx \frac{1}{2^{128}}\;(\mathrm{extremely}\;\mathrm{small})$$

Thus, the unicast key negotiation process provides strong confidentiality guarantees for data exchanged between the STA and AP.

**Theorem** **2.** *If the unicast key $K_{\mathrm{unicast}}$ is securely negotiated and used for encryption, then no attacker can successfully decrypt the data without the correct key.*

**Proof.**  Let $C_{\mathrm{data}}$ represent an encrypted message intercepted by an attacker. Without knowing the unicast key $K_{\mathrm{unicast}}$, the attacker cannot decrypt the message. The unicast key negotiation process ensures that the key is shared only between the STA and AP. Therefore, the probability that an attacker can successfully decrypt the message is:

(9) $$P_{\mathrm{decrypt}}=\frac{1}{2^{n}}\;\mathrm{for}\;\mathrm{an}\;\mathrm{AES}\;\mathrm{key}\;\mathrm{of}\;\mathrm{size}\;n.$$

Thus, the unicast key negotiation process ensures that the probability of a successful attack is negligible. □

#### 3.4.3. Multicast Key Distribution

The AP generates and distributes a multicast key $K_{\mathrm{multicast}}$ to the STA to protect multicast traffic, ensuring that only authenticated devices can access the multicast data.

The encryption of multicast traffic is described by:

(10) $$C_{\mathrm{multicast}}={\mathrm{Enc}}_{K_{\mathrm{multicast}}}\left(P_{\mathrm{multicast}}\right)$$

where $P_{\mathrm{multicast}}$ represents the plaintext multicast message, and $C_{\mathrm{multicast}}$ represents the encrypted multicast message.

Let $P_{\mathrm{decrypt}}$ represent the probability that an unauthorized device can decrypt multicast traffic. Since only authenticated devices possess the correct multicast key, the probability is:

(11) $$P_{\mathrm{decrypt}}=0\;(\mathrm{assuming}\;\mathrm{secure}\;\mathrm{distribution}\;\mathrm{of}\;\mathrm{multicast}\;\mathrm{key})$$

Thus, multicast traffic is effectively protected from unauthorized access.

**Theorem** **3.** *If the multicast key $K_{\mathrm{multicast}}$ is securely distributed to authenticated devices, then only those devices can decrypt and access the multicast traffic.*

**Proof.**  Let an unauthorized device intercept multicast traffic. Without access to the multicast key $K_{\mathrm{multicast}}$, the device cannot decrypt the traffic. Therefore, the security of multicast traffic is guaranteed, and the probability of unauthorized decryption is zero:

(12) $$P_{\mathrm{decrypt}}=0\;(\mathrm{secure}\;\mathrm{key}\;\mathrm{distribution})$$

Thus, the multicast key distribution process ensures that only authenticated devices can decrypt the multicast traffic. □

#### 3.4.4. Linking Theoretical Analysis to Implementation

The preceding theoretical analysis established the cryptographic strength of WAPI’s core security components. This section highlights how these mechanisms were implemented in practice and verifies that the assumptions made in the equations are aligned with real-world deployment.

In the implementation, both the STA and AP were provisioned with SM2 certificates signed by a trusted certificate authority. During the authentication process, certificate exchange and validation were executed through the WAPI handshake. These operations were implemented using cryptographic libraries such as GMSSL. Verification results directly impacted session establishment—if either party’s certificate was invalid, the connection was denied. This matched the assumption in the probability model that only valid certificates yielded successful authentication.

After successful certificate verification, the STA and AP negotiated a unicast session key using an authenticated key exchange protocol. In practice, this involved a secure exchange of nonces and key materials, with the session key derived using a cryptographic hash function. The negotiated key was used for symmetric encryption (e.g., AES-128) and stored in protected memory. The theoretical model assumed that brute-force attacks were infeasible, which was supported by the implementation—no key was transmitted in plaintext, and the key length conformed to modern security standards.

The multicast key was generated by the AP and distributed securely to authenticated STAs using encrypted WAPI control frames. These frames were protected using the established unicast session key. Only devices that had completed the full authentication process could decrypt and install the multicast key, ensuring that unauthorized devices could not access the multicast data. This reflected the assumption that the probability of unauthorized decryption was zero, provided the key distribution was securely implemented.

## 4. Testing and Verification

### 4.1. Testing Environment

**Figure 3:**
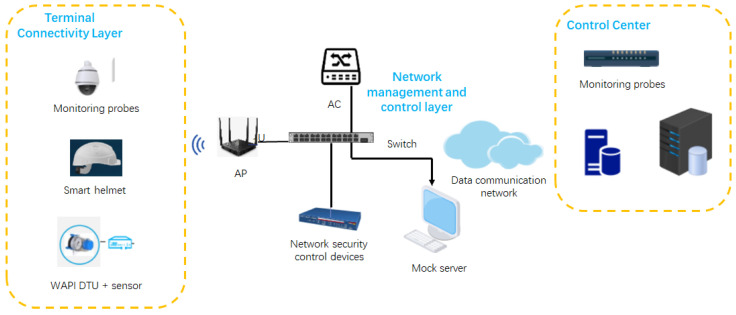
Network topology diagram in the smart factory.

The implementation of the WAPI protocol was tested in the context of a smart-factory environment. Figure 3 outlines the network topology, highlighting the connections and data flow among various devices and system layers, ensuring robust and secure data transmission. The testing environment was designed to comprehensively evaluate the effectiveness of WAPI in ensuring secure communications across different layers of the smart-factory system.

The Terminal Connectivity Layer consisted of various monitoring probes used for real-time environmental and device status data collection. It also included smart helmets equipped with sensors that ensured operator safety via continuous wireless data transmission. These terminals were connected through WAPI-enabled devices, focusing on the reliability and stability of their secure communication.

The Network Management and Control Layer was responsible for managing network communications securely. It included an access point (AP) for connecting devices, an Access Controller for authentication and policy enforcement, and additional security devices for threat detection and prevention. Switches were used to manage the flow of data between network components, while a mock server was implemented to test the overall functionality of the network. This layer was instrumental in verifying the capability of WAPI to securely handle network access and management tasks in real time.

The Control Center integrated monitoring probes to collect data and servers for data storage and analysis. It was configured to generate reports that were crucial for decision-making processes, ensuring that the data collected from the network were reliable, secure, and useful for operational purposes. This layer played a key role in verifying the successful integration of monitoring and analysis systems within the WAPI framework.

The major devices used in the testbed are listed in Table 1. The deployed system included a WAPI AS (AC 220 V) for authentication, five WAPI APs (DC 12 V) for wireless access, and a Secure Gateway (AC 220 V) for edge protection. Eight Hikvision cameras (DC 12 V) provided video surveillance, ten smart helmets (battery/DC) tracked worker locations, and twelve WAPI sensors (DC 12 V) collected industrial data, ensuring secure and automated monitoring.

**Figure 4:**
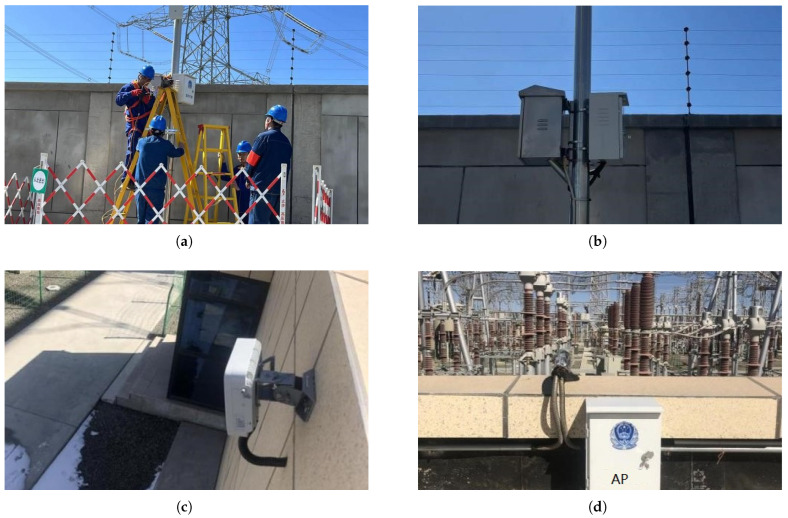
Diagram of the installation for AP devices supporting the WAPI protocol: (**a**) Workers installing the AP device on a surveillance pole. (**b**) The appearance of the surveillance pole with the AP device. (**c**) The appearance of exterior wall with the AP installation. (**d**) Rooftop installation appearance with AP.

The installation positions of AP devices primarily fell into two categories, on surveillance poles and on buildings, as illustrated in Figure 4. Firstly, for the devices installed on surveillance poles, power was drawn from the existing convergence monitoring box within the station. Communication fibers were led from the nearest corridor, with PE pipes buried to the location of the surveillance pole. The AP was installed at a height of 3.5 m. Secondly, for the devices installed on buildings, power was obtained from the AC distribution cabinet in the small room, with power lines and optical cables routed from the station corridor through the rooftop of the small room to the pre-buried steel pipes in the corridor.

The testing environment was designed to ensure that the WAPI protocol could meet the security requirements of a smart-factory environment, providing reliable, secure, and efficient communication between various devices and system layers.

### 4.2. Equipment Configurations

#### 4.2.1. Network Cameras

A Hikvision network camera was integrated into the WAPI network via a WAPI CPE terminal. This scenario simulated the conversion of wired terminals to operate wirelessly within a WAPI environment.

The configuration process for the implementation of the WAPI protocol in the testing environment was conducted in several steps to ensure successful integration and functionality of the devices. The following steps outline the configuration details.

The first step involved applying for WAPI terminal certificate verification on the AS. This was crucial to ensure that all devices connecting to the WAPI-enabled network were properly authenticated, thereby maintaining the security of the communication system.

**Figure 5:**
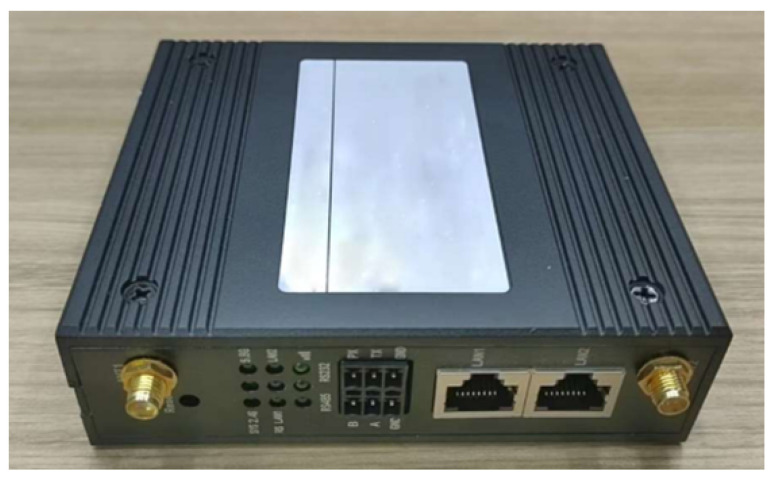
XH-CPE510 device.

The device was configured by powering it on and importing a pre-applied WAPI certificate. This certificate allowed the device to authenticate and securely communicate over the WAPI network. The device was set to client mode, and its LAN1 port was configured in NAT mode. After the configuration, the CPE successfully acquired an IP address, specifically 192.168.1.3, confirming its connection to the network. The proper configuration of the CPE device XH-CPE510 was essential for its role as a client device within the WAPI network. The XH-CPE510 device is shown in Figure 5.

This configuration allowed the camera to securely transmit video data over the WAPI-enabled network and ensured that it could communicate effectively with other network components.

#### 4.2.2. Smart Helmets

Smart helmets can directly connect to existing WAPI networks via built-in WAPI chips, simulating scenarios for adding new WAPI terminals. The configuration process for enabling secure network connectivity and data transmission of the smart helmet involved several key steps, which are detailed below.

First, the process was initiated by applying for the WAPI terminal certificate from the WAPI AS. This step was crucial to ensure that the smart helmet could be authenticated and securely communicate within the WAPI-enabled network.

Then, the smart helmet was activated, and the acquired WAPI certificate was imported. Importing the certificate allowed the helmet to establish a secure connection with the network, enabling secure data transmission.

After that, the smart helmet was connected to the AP to initiate data transmission. Establishing this connection ensured that data collected by the smart helmet could be transmitted to the designated server without interruptions.

Subsequently, the smart helmet was configured to send data to a designated server address. This configuration ensured that data routing and management were conducted properly, allowing efficient data processing and analysis.

Finally, the data uploaded by the smart helmet were monitored using the simulated server interface. The successful transmission of data was verified, confirming that the smart helmet was able to securely upload data to the designated server address as intended.

#### 4.2.3. WAPI DTU and Sensor

**Figure 6:**
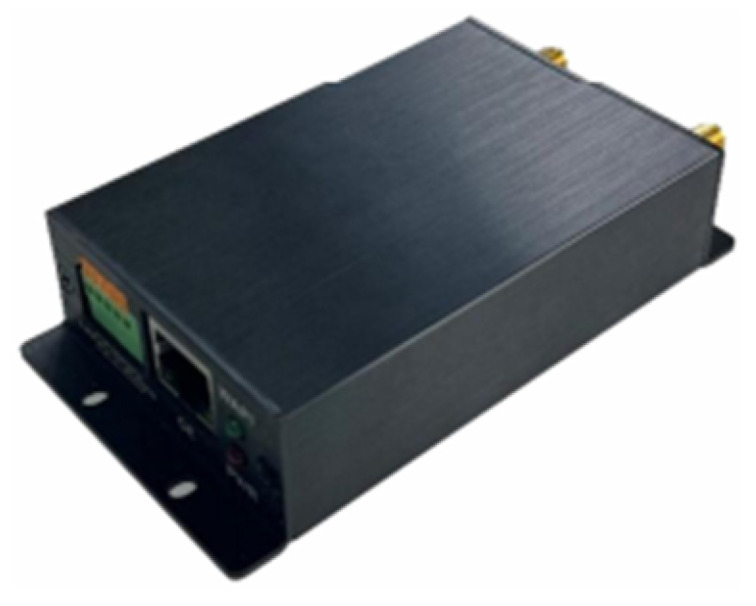
DTU device.

Temperature and humidity sensors were connected to the WAPI network via a Data Transmission Unit (DTU) terminal shown in Figure 6, simulating the integration of non-IP devices into the WAPI environment.

Firstly, the verification of the WAPI terminal certificate was conducted on the AS. The process began by logging into the AS and initiating the verification procedure for the WAPI terminal certificate. This step was crucial in ensuring that all connected devices were properly authenticated, thereby maintaining network security.

Secondly, the DTU was configured by specifying its operating mode, configuring its network port address, and importing the necessary WAPI certificate. These configurations allowed the DTU to communicate securely within the WAPI network. Proper configuration of the DTU ensured that it could establish a reliable and secure connection with other devices in the network.

Then, the sensor was configured and connected to the DTU via a serial port connection. After connecting the sensor to the DTU, the sensor was powered on, allowing it to begin transmitting data. This setup facilitated the collection of environmental data, which was crucial for monitoring purposes within the smart-factory environment.

In the end, the temperature and humidity data uploads were monitored through the simulated server’s interface. The successful transmission of sensor data was verified, confirming that the sensor was properly connected and that the DTU was functioning as expected. This step ensured that the collected environmental data could be reliably transmitted and processed within the WAPI-enabled network.

The configuration process involved verifying the WAPI terminal certificate, configuring the DTU and connecting environmental sensors, and monitoring data uploads via the simulated server. These steps were essential in establishing a secure and functional network, ensuring reliable sensor data collection and transmission within the WAPI framework.

Through our test, the sensors demonstrated 99% uptime during the one-month testing period. The WAPI network handled data from up to 50 sensors simultaneously without any performance degradation, highlighting the protocol’s reliability and efficiency.

### 4.3. Testing Results

#### 4.3.1. Signal Measurement Coverage Test

**Figure 7:**
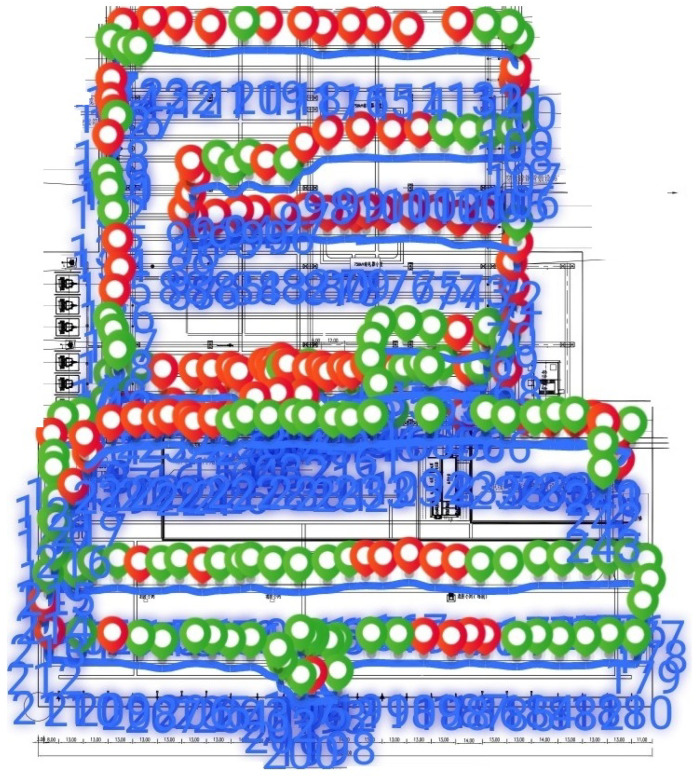
The factory’s WAPI signal measurement results. Green dots represent sampling points with a signal strength greater than −65 dBm, while the red dots indicates sampling points with a signal strength lower than −65 dBm.

A total of 243 test points were selected within the factory to conduct signal coverage strength tests, as illustrated in Figure 7.

From the results within the measurement range, it was observed that the signal coverage was good overall without any blind spots; however, there were areas with weak coverage. There were 170 points (70%) with good signal strength (RSSI higher than −65 dBm) and 73 points (30%) with poorer signal strength (RSSI lower than −65 dBm). The highest measured value was −40.5 dBm, and the lowest was −74 dBm. Seventy percent of the measurement points were classified as having good signal strength, and the signal strength at all measurement points was not lower than −75 dBm. Points with excellent signal strength (RSSI higher than −45 dBm) were identified. Although there were few areas with weaker signal strength (RSSI below −65 dBm), there were no evident blind spots.

#### 4.3.2. Throughput Testing Under Multi-Network Interference Conditions

In this experiment, we tested the throughput of an indoor WAPI AP device within a server room environment under multi-network interference conditions. The test involved measuring the platform-to-end throughput with six connected cameras, all of which were in an online state. Despite the presence of interference from multiple networks, the results indicated that the throughput for any test point was consistently above 10 Mbps in the 2.4 GHz frequency band and above 100 Mbps in the 5 GHz frequency band. The detailed test results are presented in Table 2 and Table 3.

#### 4.3.3. Latency Testing Under Multi-Network Interference Conditions

In this experiment, we tested the latency of an indoor WAPI AP device within a server room environment under multi-network interference conditions. The test involved measuring the platform-to-end latency with six connected cameras, all of which were in an online state. Despite the presence of interference from multiple networks, the results indicated that the latency for any test point was consistently below 5 ms in both the 2.4 GHz and 5 GHz frequency bands. The detailed test results are presented in Table 4.

#### 4.3.4. WAPI Authentication Server Performance

Due to the limitations of conducting on-site testing for the WAPI Authentication Server (AS), we commissioned a third-party organization specializing in WAPI to perform a comprehensive evaluation of the TH-AS2001 WAPI server. A detailed technical report was provided following the testing process.

Table 5 summarizes the test items that were performed, all of which were successfully passed. In particular, in the Certificate Authentication Performance Test, the server demonstrated the ability to handle 377 authentications per second, indicating a high level of efficiency and performance.

### 4.4. Visualization Platform for IoT Devices’ Control

**Figure 8:**
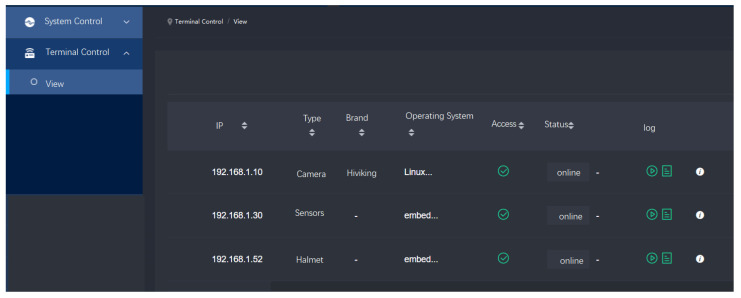
The visualization of terminal control platform.

We developed a comprehensive visualization platform that allowed for the efficient management of newly integrated devices. The platform provided an intuitive interface to oversee and control the connected devices, simplifying network management. As illustrated in Figure 8, the camera, various sensors, and the smart helmet were visible through the interface. These devices had successfully completed the WAPI authentication process and were integrated into the network, where they operated as intended. In the visualized platform, the IP address, type, brand, operating system, status, and logs of the devices were displayed.

The visualization platform enabled real-time monitoring and management of these devices, ensuring seamless network operation, enhanced security, and improved reliability of the overall system. This capability played a crucial role in promptly identifying and addressing any issues, thereby maintaining optimal performance of the connected devices within the WAPI-enabled environment.

**Figure 9:**
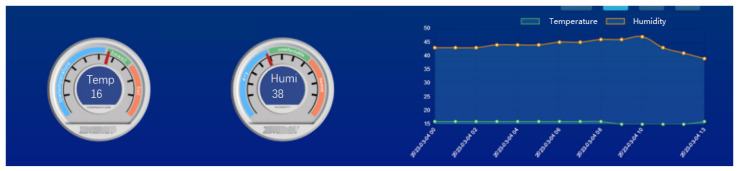
Temperature and humidity sensors’ monitor platform.

In addition, the sensors maintained stable connections to the WAPI network, with data transmission occurring in real time. The average data packet size was 512 bytes, and the transmission interval was 10 s. No data loss was recorded, ensuring the accuracy of the transmitted data. The visualization platform is shown in Figure 9, displaying two distinct sensor readings. The first reading indicates a temperature value of 16, while the second reading shows a humidity level of 38. The data are presented in a clear and concise manner, with each measurement labeled accordingly as “Temp” and “Hum”. The orange curve adjacent to the numerical values represents the trend in humidity variation over time, while the green curve illustrates the corresponding trend in temperature changes.

The platform’s basic functions included device registration, real-time status monitoring, and alert management. The device registration function allowed newly added IoT devices to be seamlessly incorporated into the network, ensuring that all devices were authenticated through the WAPI process before being granted access. The real-time status monitoring capability enabled operators to visually track the operational state of each connected device, providing information on metrics such as signal strength, data transmission rates, and device status. This continuous visibility ensured that any anomaly could be detected at an early stage, reducing the potential for network failures.

In addition to monitoring, the visualization platform also supported various control functions, enabling remote configuration of devices and facilitating network maintenance tasks. Operators could adjust sensor parameters, reset devices, and schedule routine maintenance activities directly from the interface. Furthermore, the platform provided an alert system that notified administrators in case of any detected security threats, abnormal device behavior, or connection loss. By offering these features, the platform not only enhanced the overall management efficiency of the network but also significantly improved the security posture of the system, ensuring that critical data generated by the IoT devices remained protected at all times.

The visual representation of devices and their interactions within the network also helps in understanding the overall system architecture and the relationships between different components. By visualizing the data flow from devices like cameras and sensors to the network control units, administrators are able to gain insights into the system’s functionality and optimize resource allocation accordingly. This level of control and transparency is essential in environments such as smart factories, where reliability, security, and efficiency are of utmost importance for maintaining production continuity and ensuring the safety of personnel.

### 4.5. Discussion

In this section, we discuss the findings of our research, focusing on three main aspects: the security and usability of WAPI in high-security environments such as smart factories, the support and verification for various types of devices, and the establishment of a visualization platform for effective data management.

#### 4.5.1. Security and Usability of WAPI in Smart Factories

The WAPI protocol demonstrated high levels of security and usability within the smart-factory setting. Our experimental results indicated that WAPI effectively prevented unauthorized access and ensured the integrity and confidentiality of data transmission. The protocol’s robust security mechanisms, such as the use of a dynamic authentication key and a strong encryption algorithm, rendered it particularly suitable for high-security environments.

To clarify the rationale for adopting the WAPI protocol in our system, we conducted a comparative analysis with WPA3, as presented in Table 6. While WPA3 is widely used in general-purpose consumer environments such as public Wi-Fi and home networks, WAPI offers distinctive advantages tailored to the stringent security and operational requirements of industrial scenarios like smart factories and power grids. Key differences lie in authentication mechanisms, encryption algorithms, regulatory support, and the ability to operate in environments with limited or offline access to certificate authorities. These characteristics make WAPI particularly well suited for mission-critical and infrastructure-sensitive applications.

#### 4.5.2. Security Threat Models and WAPI Defense Capabilities

To evaluate the robustness of WAPI, we analyzed its defenses against four common wireless attack vectors: spoofing, replay, man-in-the-middle (MITM), and denial-of-service (DoS).

Spoofing (impersonation): WAPI mandates mutual authentication using digital certificates based on the SM2/SM4 elliptic curve algorithm. Both APs and terminals must present valid certificates issued by a trusted Authentication Service Entity (ASE), effectively preventing unauthorized impersonation due to the computational infeasibility of forging such certificates.

Replay attacks: WAPI incorporates unique session identifiers, per-message sequence counters, and timestamp validation to detect and discard stale or duplicated messages, thereby maintaining the freshness and integrity of communications.

MITM attacks: Through a three-phase certificate authentication protocol, WAPI ensures that all session participants are cryptographically verified. Any tampering or unauthorized intervention is detected by the ASE, preserving message authenticity and confidentiality.

DoS attacks: while WAPI lacks dedicated anti-DoS mechanisms, it employs AP-level filtering and early packet integrity checks to mitigate flooding and malformed traffic, reducing unnecessary resource consumption during authentication.

In summary, WAPI’s certificate-based architecture, session-aware message validation, and early-stage filtering provide comprehensive defense against key wireless threats, supporting its suitability for industrial and IIoT environments.

#### 4.5.3. Support and Verification for Diverse Devices

Our proposed solution provided comprehensive support for a wide range of devices, including those with advanced operating systems and those without any operating system. Through rigorous testing and verification, we confirmed that the WAPI protocol could be effectively implemented on these devices, ensuring secure and reliable network access.

However, we encountered challenges in integrating the WAPI protocol with certain environmental monitoring equipment and hardware that lacked the necessary support. To address this issue, we plan to collaborate with manufacturers to open up interfaces and enable the adoption of the WAPI protocol on these devices. This would further expand the applicability of our solution and enhance the security of data transmission in all aspects of the smart factory.

#### 4.5.4. Energy Efficiency Considerations

Although direct energy consumption measurements were not conducted in this study, WAPI exhibits favorable energy characteristics in practice. Once a secure session is established, the protocol’s low-overhead frame structure and efficient reuse of session keys reduce the computational cost per packet, leading to lower energy consumption during sustained data transmission.

Real-world tests further confirmed that WAPI-enabled devices operated stably with minimal retransmissions or authentication retries, indirectly reflecting its communication and energy efficiency. These traits make WAPI suitable for deployment in embedded, mobile, or battery-powered devices.

To further improve WAPI’s applicability in energy-constrained environments such as IIoT terminals, several optimizations can be considered. These include extending device sleep intervals through coordinated wake-up scheduling between access points and terminals, reducing unnecessary handshakes by adaptively adjusting authentication intervals based on device activity, and employing lightweight session key caching to avoid frequent full authentication exchanges. Combined with selective use of hardware cryptographic acceleration, these enhancements can significantly reduce energy consumption while maintaining WAPI’s strong security guarantees.

#### 4.5.5. Intuitive and Cutting-Edge Visualization Platform

To better manage and monitor the data transmitted by various devices in the smart factory, we established a visualization platform. This platform allowed for real-time monitoring, analysis, and visualization of data, providing valuable insights into the operational status of the factory. The visualization platform was designed to be user-friendly and adaptable to different data formats, ensuring that it could be easily integrated into the existing factory management system.

The platform not only facilitates efficient data management but also contributes to the rapid detection of anomalies and potential security breaches. By visualizing the data, operators are able to quickly identify the issues and take appropriate actions, thereby improving the overall efficiency and security of the smart factory.

## 5. Conclusions

In conclusion, our study validated the efficacy of the WAPI protocol for secure and efficient data transmission in smart-factory environments. Moreover, we developed a robust visualization platform that facilitated real-time monitoring and management of networked devices. Our findings underscored WAPI’s potential as a cornerstone technology for secure industrial IoT applications, ensuring reliable connectivity and robust data security for smart-factory networks.

## Figures and Tables

**Table 1:** Devices used in the testing environment.

Device Name	Quantity	Power Supply	Remarks
WAPI AS	1	AC 220 V	Core WAPI AS node
WAPI AP	5	DC 12 V	WAPI wireless access
Secure Gateway	1	AC 220 V	Edge protection
Hikvision Camera	8	DC 12 V	Video surveillance
Smart helmets	10	Battery/DC	Worker location
WAPI sensor	12	DC 12 V	Industrial data upload

**Table 2:** Throughput measurement at test points for uplink.

Test Point	WAPI-2.4G (Mbps)	WAPI-5G (Mbps)
1	25.8	219
2	28.9	247
3	16.7	215
4	27.6	230
5	24.7	230
6	20.0	236

**Table 3:** Throughput measurement at test points for downlink.

Test Point	WAPI-2.4G (Mbps)	WAPI-5G (Mbps)
1	16.3	174.9
2	18.8	206.8
3	18.2	154
4	19.5	189
5	16.7	178
6	20.0	192

**Table 4:** Latency measurement at test points.

Test Point	WAPI-2.4G (ms)	WAPI-5G (ms)
1	3.37	3.48
2	3.37	3.56
3	3.36	3.48
4	3.71	3.34
5	3.38	3.78
6	3.69	3.25

**Table 5:** WAPI protocol test results [15].

Reference Section	Test Item	Result
7.3.3.1 X.509 V3 Certificate Management Test	X.509 v3 Certificate Management Test	passed
7.3.2 WAPI Port Number Test	Certificate Authentication and Key Management	passed
7.3.3.2 X.509 Protocol Process	Certificate Authentication and Key Management	passed
7.3.3.4 MAC Address Binding	MAC Address Binding Function Test	passed
7.3.3.7 Roaming Test	Certificate Authentication (Access Point AS roaming)	passed
7.3.4 WAPI Protocol Integrity	WAPI Subtype field in Auth Request WAPI Version field in Auth Request WAI Type field in Auth Request	passed
6.5.1 Authentication Performance Testing	Certificate authentication performance test	377/S

**Table 6:** Comparison between WAPI and WPA3.

Feature	WAPI	WPA3
Authentication	Certificate-based (PKI)	Password-based (SAE)
Encryption algorithm	ECC, SM4, AES	AES-GCMP
Regulatory origin	Chinese National Standard	IEEE Standard
Offline/local CA support	Supported	Not supported
Mutual authentication	Mandatory	Optional
Industrial adaptation	Smart grids, power systems	Public Wi-Fi, consumer devices

## Data Availability

Data are contained within the article.

## Abbreviations

The following abbreviations are used in this manuscript:

IoT	Internet of Things
WAPI	Wireless LAN Authentication and Privacy Infrastructure Protocol
MIC	Message Integrity Code
STA	Terminal device
AP	Access point
AS	Authentication Server
CPE	Customer-Premises Equipment
PKI	Public Key Infrastructure
Modbus RTU	Modbus Remote Terminal Unit
MITM	Man in the middle
DoS	Denial of service
DTU	Data Transmission Unit
